# Identification of long-range chromatin interactions between HTLV-1 and the host genome

**DOI:** 10.1186/1742-4690-12-S1-O10

**Published:** 2015-08-28

**Authors:** Hiroko Yaguchi, Anat Melamed, Aileen Rowan, Lucy Cook, Yorifumi Satou, Charles R M Bangham

**Affiliations:** 1 Imperial College London, London, W2 1PG, UK; 2 Kumamoto University, Kumamoto, 860-0811, Japan

## 

Human T-lymphotropic virus type I (HTLV-1) integration is known to be weakly biased towards genomically active regions but can occur throughout the genome. We have shown that there are of the order of 10^4 HTLV-1-infected T-cell clones in each individual, each clone defined by a unique genomic integration site of the single-copy HTLV-1 provirus. HTLV-1 dysregulates many host genes by expressing a transcriptional transactivator, Tax, but it remains unknown whether HTLV-1 also alters host gene expression by insertional mutagenesis. In recent work (Satou et al, submitted) we have shown that HTLV-1 encodes a functional binding site for CCCTC binding factor (CTCF), a known mediator of chromatin looping. We hypothesize that the HTLV-1 provirus forms CTCF-mediated chromatin loops with the flanking host genome and dysregulates the expression of host genes. To test this hypothesis, we performed a circular chromatin conformation capture assay followed by high-throughput sequencing (4C-seq) which enables high resolution screening of physical long range interactions between chromosomes. Using the proviral CTCF site as bait, we have detected long-range interactions between the proviral and the host genome which suggest the formation of novel chromatin looping in clones of HTLV-1-infected T cells from both cases of ATL and from non-malignant cases of HTLV-1 infection. Chromosome conformation capture using specific primers (3C) confirmed clone-specific interactions. We are now testing whether these long-range interactions are associated with clone-specific alterations in the expression of host genes.

